# A Systematic Review on Re-irradiation with Charged Particle Beam Therapy in the Management of Locally Recurrent Skull Base and Head and Neck Tumors

**DOI:** 10.14338/IJPT-20-00064.1

**Published:** 2021-06-25

**Authors:** Mauricio E. Gamez, Samir H. Patel, Lisa A. McGee, Terence T. Sio, Mark McDonald, Jack Phan, Daniel J. Ma, Robert L. Foote, Jean-Claude M. Rwigema

**Affiliations:** 1 Radiation Oncology, The Ohio State University – The James Cancer Center, Columbus, OH, USA; 2 Radiation Oncology, Mayo Clinic, Phoenix, AZ, USA; 3 Radiation Oncology, Winship Cancer Institute of Emory University, Atlanta, GA, USA; 4 Radiation Oncology, MD Anderson Cancer Center, Houston, TX, USA; 5 Radiation Oncology, Mayo Clinic, Rochester, MN, USA

**Keywords:** proton therapy, carbon ion therapy, particle beam therapy, charged particle therapy, re-irradiation, skull base and head and neck cancer

## Abstract

**Purpose:**

To evaluate the clinical outcomes and treatment related toxicities of charged particle-based re-irradiation (reRT; protons and carbon ions) for the definitive management of recurrent or second primary skull base and head and neck tumors.

**Materials and Methods:**

The Preferred Reporting Items for Systematic Reviews and Meta-Analyses (PRISMA) guidelines were applied for the conduct of this systematic review. Published work in English language evaluating the role of definitive charged particle therapies in the clinical setting of reRT for recurrent or second primary skull base and head and neck tumors were eligible for this analysis.

**Results:**

A total of 26 original studies (15 protons, 10 carbon ions, and 1 helium/neon studies) involving a total of 1,118 patients (437 with protons, 670 with carbon ions, and 11 with helium/neon) treated with curative-intent charged particle reRT were included in this systematic review. All studies were retrospective in nature, and the majority of them (n=23, 88 %) were reported as single institution experiences (87% for protons, and 90% for carbon ion-based studies). The median proton therapy reRT dose was 64.5 Gy (RBE 1.1) (range, 50.0 – 75.6 Gy ), while the median carbon ion reRT dose was 53.8 Gy (RBE 2.5 – 3.0) (range, 44.8 – 60 Gy ). Induction and/or concurrent chemotherapy was administered to 232 (53%) of the patients that received a course of proton reRT, and 122 (18%) for carbon ion reRT patients. ReRT with protons achieved 2-year local control rates ranging from 50% to 86%, and 41% to 92% for carbon ion reRT. The 2-year overall survival rates for proton and carbon ion reRT ranged from 33% to 80%, and 50% to 86% respectively. Late ≥ G3 toxicities ranged from 0% to 37%, with brain necrosis, ototoxicity, visual deficits, and bleeding as the most common complications. Grade 5 toxicities for all treated patients occurred in 1.4% (n= 16/1118) with fatal bleeding as the leading cause.

**Conclusions:**

Based on current data, curative intent skull base and head and neck reRT with charged particle radiotherapy is feasible and safe in well-selected cases, associated with comparable or potentially improved local control and toxicity rates compared to historical reRT studies using photon radiotherapy. Prospective multi-institutional studies reporting oncologic outcomes, toxicity, and dosimetric treatment planning data are warranted to further validate these findings and to improve the understanding of the clinical benefits of charged particle radiotherapy in the reRT setting.

## Introduction

Head and neck cancers pose a global clinical challenge, with an annual incidence of more than 650,000 cases and 330,000 deaths [1]. The majority of patients present with squamous cell carcinomas of the nasopharynx, oropharynx, oral cavity, hypopharynx, or larynx, although many other histologies and tumor subsites are clinically recognized. The variably aggressive and diverse anatomic and biological behaviors contribute to the complexity of disease management and difficulty in achieving optimal treatment outcome. Consequently, even after curative intent therapy, recurrence is quite common despite advances in multimodality management of head and neck cancers [2, 3].

Salvage surgery is considered the first-line therapy, for the majority of previously irradiated recurrent cases, with the exception of recurrent nasopharyngeal cancer were re-irradiation (reRT) remains the first choice of treatment. However, not all patients are candidates for salvage surgery as they may be medically unfit for surgery, salvage surgery may be unreasonably morbid or unable to achieve a complete resection, or patients may decline surgery [4, 5]. Given limited salvage options, reRT has historically played a role in the management of recurrent head and neck cancer, but posed quite a challenge due to lower chances of disease control coupled with the increased risk for severe toxicities [6]. ReRT with modern techniques such as intensity modulated (IMRT) and stereotactic body radiotherapies (SBRT) has led to improved tumor control with less severe toxicity and improved quality of life when compared to re-RT with 2D and 3D conformal radiotherapies [7, 8].

Charged particle radiotherapy including proton (PT) and carbon ion radiotherapy (CT) are frequently considered for reRT due to their more favorable radiation dosimetry, which can often improve normal tissue sparing of organs at risk from additional radiation. In previously irradiated patients, the therapeutic window is often narrow, and there may be significant risks associated with the high cumulative radiation dose to normal tissues, which can be mitigated through improved sparing of these normal non-target tissues. More data on the efficacy and comparative effectiveness of charged particle therapy in the setting of re-irradiation are needed to better understand the most appropriate application of this limited resource and to guide further clinical investigation. This review evaluates the clinical outcomes and treatment related toxicities of charged particle reRT for the definitive management of recurrent or second primary skull base and head and neck neoplasms.

## Materials and Methods

The Preferred Reporting Items for Systematic Reviews and Meta-Analyses (PRISMA) guidelines were applied for the conduct of this systematic review [9]. Published work in English language evaluating the role of charged particle reRT in the setting of recurrent or second primary head and neck neoplasms that have previously undergone at least one prior course of RT, and with charged particle reRT delivered overlapping with the prior irradiated field were eligible for this analysis.

A broad search was initially performed, and included the following databases: PubMed, Medline, Embase, Cochrane, Google Scholar, Ovid, Scopus, as well as publications identified from references of previously published articles, and articles known to the authors. The initial search sought to comprehensively identify all published articles addressing the topic by using the following legends: (Proton(s), Proton Radiation Therapy, Proton Therapy, Proton Beam Therapy, Proton Beam, Charged Particle, Charged Particle Therapy, Particle Therapy, and Carbon ion (s)), (Reirradiation, Re-irradiation, Reradiation, Re-radiation, Radiation Retreatment, Radiation Re-treatment, Retreat, Re-treat, reRT, and re-RT), (Recurrent Cancer, Recurrent Disease, Secondary Cancer, Secondary Malignancy, Salvage Treatment), and (head and Neck, Head and Neck Disease Site). Disease site-specific searching criteria included: Pharynx, Nasopharynx, Oropharynx, Larynx, Hypopharynx, Oral Cavity, Oral Cancer, Salivary Gland, Parotid, Parotid Gland, Skin Cancer, Scalp, Sinonasal, Sino-nasal, Paranasal Sinuses, Para-Nasal Sinuses, Sinuses, Nasal, Nasal Cavity, Base of Skull, Skull Base, Scalp, Orbit, Eye, Ocular, Thyroid, and Thyroid Gland.

**Figure 1.**
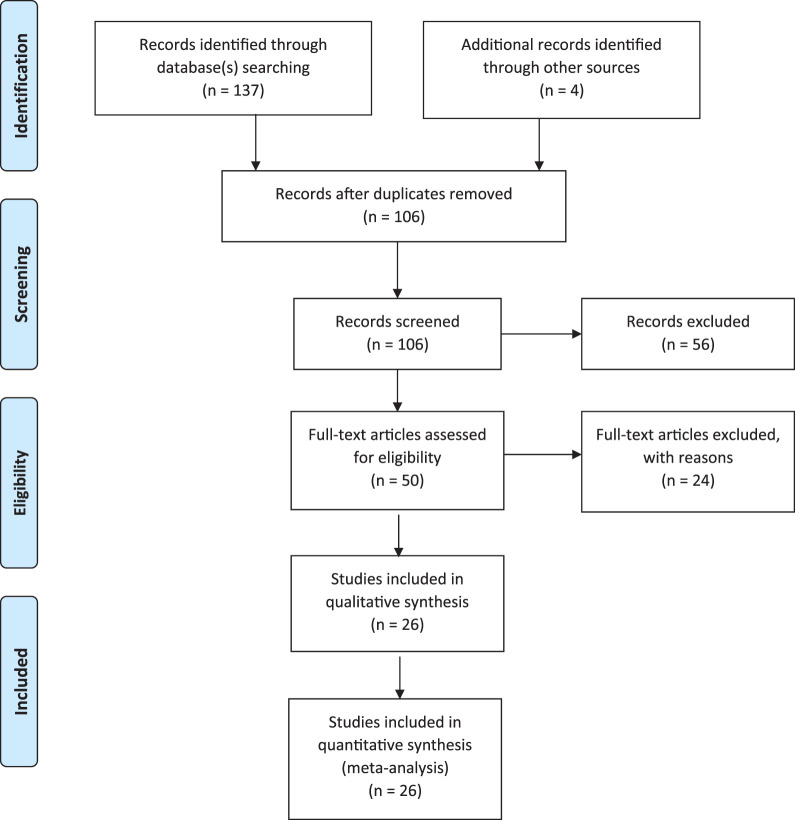
PRISMA flow diagram of systematic searches and selection.

No date restrictions were employed in our planned search. All identified published articles through July 2020 were included in the initial evaluation. All articles were screened by 2 authors: Mauricio E. Gamez, and Jean-Claude Rwigema. A total of 141 studies were identified based on our initial search criteria: 137 from the database(s) search, and 4 additional articles were identified through other sources (Fig. 1). Publications of the same study population from the same institution or group of investigators and/or series that used charged particle reRT as a palliative treatment option were excluded. After these reports were removed, the remaining 106 eligible items were screened based on the previously discussed search criteria, and a total of 56 records were further excluded. In addition, articles without any well specified clinical endpoints (ie. local control, survival, or side effects) of charged particle radiotherapy were also excluded. Of the 50 remaining publications, review articles, abstracts, letters to the editor, commentaries, and studies with ≤ 5 reRT patients were excluded. Thus, 26 original studies were found to have sufficient focus and relevance to be incorporated and analyzed in this systematic review. A meta-analysis was not performed due to the heterogeneity of the reported reRT head and neck series, and the lack of consistent statistical power and value in this setting.

## Results

A total of 26 original published studies (15 protons, 10 carbon ions, and 1 helium/neon ion particle radiotherapy) involving a total of 1118 patients (437 with protons, 670 with carbon ions, and 11 patients with helium/neon ions) who were treated with curative-intent charged particle reRT were included in this comprehensive systematic review [10 – 35]. According to head and neck subsite, charged particle reRT with either protons or carbon ions was most commonly used for recurrent sinonasal, nasopharyngeal and salivary gland tumors, with squamous cell and adenoid cystic cell carcinoma as the most frequent histologies. Table 1 and 2 summarizes the different reRT studies using either proton, carbon ion or helium/neon ion therapy for the management of recurrent head and neck malignancies. All studies were retrospective in nature, and the majority (23 of 26 studies, 88 %) belonged to single institutional experiences (13 of 15 studies, or 87% for protons, and 9 of 10 studies, or 90% for carbon ions; the only helium/neon study also involved one institution). Geographically 10 (67%) of the 15 proton series were reported by the United States, 3 (20%) from Asia, and the remaining 2 studies (13%) from Europe. With respect to carbon ion radiotherapy, all studies were implemented either in Asia 5/10 (50%) or in Europe 5/10 (50%). The study series that used charged particle therapy with Helium and Neon was carried out in the Lawrence Berkeley Laboratory in the USA. The included studies reported on patients treated between 1981 – 2018, with fifty percent (n=13/26) of studies (n=7/15 protons, and n= 6/10 carbon ions) occurring in the last ten years, suggesting an increased availability and interest in the use of charged particle radiotherapy in the reRT setting.

**Table 1.** Series of proton reirradiation (reRT) of the head and neck (HN) and skull base series.

**Author [citation] study period institution [country)**	**Study type** **No. pts**	**Primary site** **histology (%)**	**Previous RT technique (%)** **median previous RT dose (range)/fractionation**
Dionisi et al. [10] 2015–2018 Proton Therapy Unit, APSS (Italy)	Retrospective, single institution 17	NPC SCC (35%) Non-keratinizing carcinoma (23%) Undifferentiated carcinoma (42%)	3D-CRT (35%) IMRT (65%) 70 Gy (60–74)/2Gy (1.8–2.5)
Hayashi et al. [11] 2009–2014 Southern Tohoku Proton Therapy Center (Japan)	Retrospective, single institution 34	Oral Cavity SCC (88%) ACC (6%) Mucoepidermoid (3%) Ameloblastic Ca (3%)	EBRT (79%) BT (15%) PBT (59%) 55 Gy (40–80)/NA
McDonald et al. [12] 2004–2014 Indiana University (United States)	Retrospective, single institution 61	Multiple HN disease sites SCC n = 32 Non-SCC n = 29	EBRT (n = 61) IORT (n = 2) Gamma Knife (n = 3) 64.6 Gy (43.2–74): SCC 66 Gy (40–75.6): Non-SCC
Linton et al. [13] 2004–2012 Indiana University (United States)	Retrospective, single institution 6/26 (23%)	Sinonasal, Nasopharynx, orbit, major/minor salivary glands–not specified for re-irradiated cases ACC (100%)	EBRT SRS Gamma Knife NR
Yu et al. [14] 2010–2016 (multi-institution^a^) PCG (United States)	Retrospective, multi-institution 27/69 (39%)	Sinonasal SCC (41%) ACC (22%) Esthesio (15%) Adeno (15%) Other (7%)	NR NR
Fan et al. [15] 2013–2018 ProCure (United States)	Retrospective, single institution 18/86 (21%)	Sinonasal, nasal cavity/ethmoid sinus (39%), other sinuses (61%) SCC (44%) ACC (17%) Esthesio (11%) Other (22%)	NR 60 Gy (27–70)
Ng et al. [16] 2000–2016 MDACC (United States)	Retrospective, single institution 15/75 (20%)	Skull base/orbit/sinonasal (53%), nasopharynx (13%), oral cavity (13%), oropharynx (7%), other (13%) SCC (27%) Non-SCC (73%)	NR 60 Gy (30–74)/2 Gy
Romesser et al. [17] 2011–2014 (multi-institution^b^) (United States)	Retrospective, multi-institution 92	Oropharynx (18%), oral cavity (13%), nasal cavity/paranasal sinuses (13%), salivary glands (12%), larynx/hypopharynx (11%), nasopharynx (10%), other (23%) SCC (56%) Non-SCC (44%)	EBRT (100%) 66 Gy (58.2–70): SCC 58.9 Gy (49.8–65.1): Non-SCC
Phan et al. [18] 2011–2015 MDACC (United States)	Retrospective, single institution 60	Oropharynx (25%), oral cavity (5%), nasopharynx (13%), larynx (2%), parotid (12%), orbit (5%), sinonasal (20%), neck/unknown primary (5%), other (13%) SCC (67%) Non- SCC (33%)	NR 60 Gy (45–72): SCC 60 Gy (30–70): Non-SCC
Azami et al. [19] 2009–2012 Southern Tohoku Proton Therapy Center (Japan)	Retrospective, single institution 6/10 (60%)	Parotid gland ACC (40%) EMC (20%) Sarcoma (10%) SCC (10%) MC (10%) Acinic cell (10%)	NR 57.5 Gy
Yang et al. [20] 2014–2018 Shanghai Proton and Heavy Ion Center (China)	Retrospective, single institution 12/51 (23%)	Multiple HN sites Sarcoma (100%)	NR NR
Dale et al. [21] 2012–2016 National Center of Oncological Hadrontherapy (CNA0) (Italy)	Retrospective, single institution 17/96 (18%)	Rhinopharynx (35%), oropharynx (18%), oral cavity (12%), brain/meninges (18%), nasal cavity (6%), larynx (6%), skin scalp/face (6%) SCC (76%) Undifferentiated carcinoma (6%) High-grade glioma (12%) Meningioma (6%)	EBRT (100%) 66 Gy (32–70)/2Gy
Marucci et al. [22] 1984–2000 Harvard Cyclotron Laboratory (United States)	Retrospective, single institution 31	Eye Uveal melanoma (100%)	PBT 70 Gy/5 fractions (90% cases)
McDonald et al. [23] 2005–2012 Indiana University (United States)	Retrospective, single institution 16	Clivus (50%), cervical spine (12%), thoracolumbar spine (19%), sacrum (19%) Chordoma (100%)	EBRT (37%) PBT (37%) SRS (25%) 75.2 (40–79.2)
Lin et al. [24] 1991–1997 Loma Linda University Medical Center (United States)	Retrospective, single institution 16	Nasopharynx (100%) NR	Conventional RT (100%) ± brachytherapy implant (n = 4. 25%) 71.8 (50–88.2)

Abbreviations: pts, patients; RT, radiotherapy; GTV, gross tumor volume; CTV, clinical target volume; PTV, planning target volume; OAR, organs at risk; F/u, follow-up; PBT, proton beam therapy; G, grade; APSS, Azienda Provinciale per i Servizi Sanitari; NPC, nasopharyngeal carcinoma; SCC, squamous cell carcinoma; 3D-CRT, 3-dimensional conformal radiation therapy; IMRT, intensity-modulated radiation therapy; SFO, single-field optimization; MFO, multifield optimization; max, maximum; CDDP, cisplatin; carbo, carboplatin; OS, overall survival; LC, local control; ACC, adenoid cystic carcinoma; Ca, cancer; EBRT, external beam radiotherapy; BT, brachytherapy; NA, not available; FOM, figure of merit; NR, not reported; LN, lymph node; ENI, elective nodal irradiation; DOC, XXXX; CR, complete remission; PR, partial remission; ORN, osteonecrosis; IORT, intraoperative radiation therapy; LF, local failure; RF, regional failure; DM, distant metastasis; CNS, central nervous system; PCG, Proton Collaborative Group; esthesio, esthesioneuroblastoma; adeno, adenocarcinoma; FFDM, freedom from distant metastasis; FFDP, freedom from distant progression; FFLR, freedom from locoregional recurrence; 3DCPT, 3-dimensional conformal proton therapy; DFS, disease-free survival; DC, XXXX; PEG, percutaneous endoscopic gastrostomy; MDACC, MD Anderson Cancer Center; PFS, progression-free survival; LRC, locoregional control; LFFS, local failure-free survival; DMFS, distant metastasis-free survival; CB, contour beam; FFM, fat-free mass; trach, tracheostomy; CSF, cerebrospinal fluid; LRPFS, locoregional progression-free survival; EMC, epithelial myoepithelial carcinoma; MC, mucoepidermoid carcinoma; IMCT, intensity-modulated carbon therapy; Cum, cumulative; D_max_, maximum dose; Eq, equivalent; SRS, stereotactic radiosurgery; BS, branch site; BID, twice a day.

a Multi-institutions: Mayo Clinic, Northwestern Medicine Chicago Proton Center, ProCure Therapy Center, Seattle Cancer Care Alliance Proton Therapy Center, University of Maryland Proton Treatment Center, California Proton Cancer Therapy Center, Willis-Knighton Cancer Center.

b Multi-institutions: Memorial Sloan Kettering Cancer Center, ProCure Proton Therapy Center, Montefiore Medical Center, Northwestern Medicine Chicago Proton Center.

**Table 1.** Extended.

**Author [citation] study period institution [country)**	**Recurrent site salvage surgery before reRT (%)** **median time to reRT (mo)** **median No. previous RT treatments (range)**	**reRT technique (%)** **median reRT dose (range)/fractionation** **treatment intent**	**Median GTV (range)** **GTV–CTV margin** **CTV–PTV margin** **OAR doses Gy (range)** **nodal irradiation**
Dionisi et al. [10] 2015–2018 Proton Therapy Unit, APSS (Italy)	Skull base (94%) Nodal (6%) 0 % 30 (11–108) 1 (1-2)	PBT active scanning SFO (76.5%) MFO (23.5%) 60 Gy (30.6–66)/2 Gy (1.8–2) Curative, n = 16 (94%) Palliative, n = 1 (6%)	15.4 cm^3^ (5–43.3) 0.5–1.5 cm 3 mm (MFO), 4 mm (SFO) Max cumulative carotid artery: 118.5 (109–129) Max optic nerve PBT: 16.5 (0.3–49) Max cumulative optic nerve: 47 (4–60) Max chiasm PBT: 25 (0.3–50) Max cumulative chiasm: 49 (5–58) Max cumulative temporal lobe: 80.8 (30.5–117) Mean inner ear PBT: 20 (3.7–61.5) Mean cumulative inner ear: 61.9 (33.7–98.6) Not performed
Hayashi et al. [11] 2009–2014 Southern Tohoku Proton Therapy Center (Japan)	Tongue (38%) Upper gingiva (26%) Lower gingiva (15%) Buccal mucosa (9%) FOM (6%) Hard palate (6%) 0 % NR NR	PBT 50 Gy (28.6–55)/2.2 Gy Curative, n = 34 (100%)	NA 3 mm 3 mm, did not extend into critical OAR NA Not performed LNs covered if involved. ENI not performed.
McDonald et al. [12] 2004–2014 Indiana University (United States)	Multiple HN disease sites 11 (34%): SCC 18 (62%): Non-SCC 17.5: SCC 32: Non-SCC 1 course (81.3%): SCC 1 course (82.8%): Non-SCC	PBT, uniform scanning 70 Gy (36–74.5): SCC 70.2 Gy (54–75.6): Non-SCC Curative, n = 61 (100%)	33 cm^3^ ( 0–161): SCC 8.9 cm^3^ ( 0–479): Non-SCC 10 mm, reduced up to 2 mm in specific cases NA Not performed
Linton et al. [13] 2004–2012 Indiana University (United States)	Skull base NR NR NR	PBT, uniform scanning 72 Gy (66–75.6) /1.8–2 Gy Curative, n = 26 (100%)	NR CTV–PTV margin: 2 mm Allowed at preference of treating physician
Yu et al. [14] 2010–2016 (multi-institution^a^) PCG (United States)	Nasal cavity (52%) Maxillary sinus (18%) Ethmoid sinus (11%) Sphenoid sinus (4%) Not specified (15%) 13 (48%) NR NR	PBT, uniform scanning or pencil-beam scanning 60 Gy (18.2–72.3)/2 Gy Curative, n = 27 (100%)	NR NR NR Nodal irradiation (8%)
Fan et al. [15] 2013–2018 ProCure (United States)	Sinonasal, nasal cavity/ethmoid sinus (39%), other sinuses (61%) 8 (44%) NR NR	PBT, 3DCPT (55%), IMPT (45%) 68 (54–76) Curative (100%)	NR CTV–PTV margin: 3–5 mm NR Nodal irradiation (6%)
Ng et al. [16] 2000–2016 MDACC (United States)	Skull Base (100%) 9 (60%) 54 NR	PBT 66 Gy (50–70)/2 Gy Curative (100%)	24 cm^3^ (9.4–45.6) NR NR Not performed
Romesser et al. [17] 2011–2014 (multi-institution^b^) (United States)	Oropharynx (15%), nasal cavity/paranasal sinuses (14%), oral cavity (17%), salivary glands (13%), nasopharynx (13%), larynx/hypopharynx (10%), other (17%) 36 (39%) 34 1 course (83%) 2 courses (14%) ≥ 3 courses (3%)	PBT, uniform scanning 66 Gy (53.1–66.2): SCC 60.1 Gy (50–66): Non-SCC Curative (100%)	CTV volume 90.8 cm^3^ (33.4–188.3): SCC 69.1 cm^3^ (69.1–26.8–138.1): Non-SCC CTV–PTV margin: 3 mm NR NR
Phan et al. [18] 2011–2015 MDACC (United States)	Oropharynx (27%), oral cavity (2%), nasopharynx (8%), larynx (3%), parotid (13%), orbit (13%), sinonasal (18%), skull base (7%), neck (10%) 22 (55%): SCC 13 (65%): Non-SCC 47.6: SCC 33.5: Non-SCC NR	PBT, passive scatter (25%), IMPT (75%) 66 Gy (50–70): Passive scatter, 66 Gy (55–70): IMPT Curative (100%)	7.1 cm^3^ (2.4–45.6): passive scatter 11.2 cm^3^ (1.0–138.1): IMPT CTV–PTV margin: 3 mm Max right optic nerve 17.5 (1.9–53.9): passive scatter Max right optic nerve 10.5 (1.8–51.7): IMPT Max left optic nerve 9.1 (0.6–72.5): passive scatter Max left optic nerve 3.2 (3.7–50.4): IMPT Max optic chiasm 6.4 (0.5–49.4): passive scatter Max optic chiasm 14.0 (0.7–53.2): IMPT Max brainstem 6.5 (0.5–52.9): passive scatter Max brainstem 14.1 (0.5–45.70: IMPT Max spinal cord 5.3 (0.4-21.1): passive scatter Max spinal cord 11.2 (0.4–42.9): IMPT Mean cochlea 12.2 (0.2–34.4): passive scatter Mean cochlea 1.5 (0.3–51.8): IMPT Mean parotid 11.5 (0.4–45.7): passive scatter Mean parotid 8.7 (0.3–49.5): IMPT Mean oral cavity 3.8 (1.4–6.2): passive scatter Mean oral cavity 8.1 (0.5–56.6): IMPT Allowed based on particular case and at preference of treating physician
Azami et al. [19] 2009–2012 Southern Tohoku Proton Therapy Center (Japan)	Parotid gland (100%) 0% NR NR	PBT 66 Gy (56–77)/2-2.2 Gy Curative (100%)	NR GTV–CTV margin: 3–5 mm CTV–PTV margin: 3–5 mm, did not extend to critical OAR NR Not performed
Yang et al. [20] 2014–2018 Shanghai Proton and Heavy Ion Center (China)	Multiple HN sites, not specified NR for proton patients NR NR	PBT (IMPT) and/or IMCT, not specified for reRT cases Not specified for reRT cases Curative (100%)	NR GTV–CTV margin: 1–3 mm NR NR
Dale et al. [21] 2012–2016 National Center of Oncological Hadrontherapy (CNA0) (Italy)	Not specified for the reRT proton patients 16 (94%) 3.4 y Not specified for the reRT proton patients	PBT 54 Gy (30–70)/2 Gy Curative (100%)	NR NR Median CumDmaxEqd2 carotid arteries: 109 (25–167) NR
Marucci et al. [22] 1984–2000 Harvard Cyclotron Laboratory (United States)	Eye 0 % 36 (8–165) All patients received a prior course of RT, except for one who received 2 prior courses	PBT 70 Gy/5 fractions (97% cases) Curative (100%)	Tumor volume 0.44 cm^3^ NA NR NA
McDonald et al. [23] 2005–2012 Indiana University (United States)	Clivus (50%), cervical spine (12%), thoracolumbar spine (19%), sacrum (19%) 8 (50%) 37 (12–129) 1 prior course (81%) 2 prior courses (12%) 4 prior courses (7%)	PBT 75.6 (71.2–79.2)/1.8–2, except for 2 pts where tumor abutted the BS and were treated with hyperfractionated RT 1.2 Gy BID Curative (100%)	71 cm^3^ (0–701) NR Doses to OAR (BS, optic chiasm, optic nerves, spinal cord, brachial plexus) reported case by case NA
Lin et al. [24] 1991–1997 Loma Linda University Medical Center (United States)	Nasopharynx (100%) 3 (19%) 34 1 prior course (100%)	Conformal PBT 62.8 (59.4–70.2)/1.8-2 Curative (100%)	NR NR Mean max surface dose BS 11.4 Gy (1.8–20) Mean 90% of BS volume <&thinsp0.5 Gy Mean 50% of BS volume 1.2 Gy Mean 10% of BS volume 2.2 Gy Mean max dose to optic chiasm 0.4 Gy (0–3.8) Mean 90% of optic chiasm volume <&thinsp0.5 Gy Mean 50% of optic chiasm volume 1.0 Gy Mean 10% of optic chiasm volume 1.8 Gy Mean max dose optic nerves 7.1 Gy (0–22) Mean 90% of optic nerves volume 0 Mean 50% of optic nerves volume 1.4 Gy Mean 10% of optic nerves volume 3.7 Gy NR

**Table 1.** Extended.

**Author [citation] study period institution [country)**	**Additional systemic therapies, (%)**	**Median age, y**	**Median F/u (mo)**	**Clinical outcomes, %**	v**PBT acute toxicities, G (%)** **PBT late toxicities, G (%)**
Dionisi et al. [10] 2015–2018 Proton Therapy Unit, APSS (Italy)	Induction chemotherapy (6%) Induction + concomitant (6%) Concomitant (CDDP) (18%) Concomitant (Carbo) (23%) No chemotherapy (47%)	58	10	Overall 1.5-y OS: 54% 1.5-y LC: 67%; After exclusion palliative case 1.5-y OS: 59% 1.5-y LC: 73%	No ≥ G3 events 23.5% ≥ G3 events Hearing impairment (18%) Fatal bleeding uncertain cause (6%)
Hayashi et al. [11] 2009–2014 Southern Tohoku Proton Therapy Center (Japan)	Weekly intra-arterial chemotherapy Median total dose of CDDP 300 mg (120–560) If residual primary tumor after 5-7 courses of intra-arterial CDDP, additional intra-arterial DOC was delivered Median total dose of DOC 48.5 (24–105)	68	25	1-y OS: 62% 2-y OS: 42% 1-y LC: 77% 2-y LC: 60% 22 pts (65%): CR 12 pts (35%): PR	G3 dysphagia (35%) G3 oral mucositis (32%) G3 radiation dermatitis (29%) G3 ORN (3%) No G4 or G5 toxicities
McDonald et al. [12] 2004–2014 Indiana University (United States)	Induction chemotherapy (3%): SCC Induction chemotherapy (3%): Non-SCC Concomitant chemotherapy (50%): SCC Concomitant chemotherapy (7%): Non-SCC	62.5: SCC 53: Non-SCC	15.2	2-y OS: 32.7% 2-y LF: 19.7% 2-y RF: 3.3% 2-y DM: 38.3%	G5 CNS (n = 1) G3 dermatitis (n = 3) G3 soft tissue/bone necrosis (n = 3) G3 mucositis (n = 2) G5 CNS (n = 1) G5 soft tissue/bone necrosis (n = 1) G3–4 CNS (n = 3) G3–4 soft tissue/bone necrosis (n = 9)
Linton et al. [13] 2004–2012 Indiana University (United States)	No concurrent systemic therapy	46	24	2-y LC: 86% 2-y OS: 57% 2-y DM: 25%	G3 ototoxicity (n = 1) G5 CNS fluid leak/meningitis (n = 1)
Yu et al. [14] 2010–2016 (multi-institution^a^) PCG (United States)	Concomitant chemotherapy (37%)	58	26	3-y OS: 76% 3-y FFDM: 47% 3-y FFDP: 32% 3-y FFLR: 34%	G3 mucositis (12%) G3 pain (6%) G3 dermatitis (4%) G3 xerostomia (3%) G3 dysphagia (3%) G3 anorexia (3%) G3 conjunctivitis (1%) G3 hearing impairment (1%) G3 nausea (1%) No ≥ G3 late toxicities occurred
Fan et al. [15] 2013–2018 ProCure (United States)	Concomitant chemotherapy (n = 9, 50%), with CDDP (n = 6, 67%), cetuximab (n = 2, 22%), other (n = 1, 11%)	< 70 (72%) ≥ 70 (28%)	23.4	2-y LC: 77% 2-y DFS: 54% 2-y OS: 66% 2-y DC: 80%	G3 dermatitis (11%) G3 dysphagia/PEG (6%) G3 mucositis (6%) G3 brain necrosis (6%) G3 facial pain (6%) No G4 or 5 toxicities related to RT
Ng et al. [16] 2000–2016 MDACC (United States)	Induction chemotherapy (7%) Concomitant chemotherapy (67%)	66	24	2-y LC: 59% 2-y RC: 78% 2-y DC: 93% 2-y PFS: 49% 2-y OS: 74%	G3 mucositis (13%) G3 dermatitis (7%) G3 ORN and dysphagia (7%) G3 glaucoma (7%) G3 trismus (7%) G3 fibrosis (7%)
Romesser et al. [17] 2011–2014 (multi-institution^b^) (United States)	Neoadjuvant chemotherapy (4%) Neoadjuvant and concurrent chemotherapy (9%) Concurrent chemotherapy (39%) Cetuximab most common regimen	63	13	1-y LRC: 75% 1-y FFDM: 84% 1-y OS: 65%	G3 mucositis (10%) G3 dysphagia (9%) G3 esophagitis (9%) G3 dermatitis (3%) ≥ G3 skin (9%) ≥ G3 dysphagia (7%) G5 bleeding (3%)
Phan et al. [18] 2011–2015 MDACC (United States)	Induction chemotherapy (8%) Concurrent chemotherapy (73%)	66: SCC 60.5: Non-SCC	13.6	1-y LRC: 81% 1-y LFFS: 68% 1-y OS: 81% 1-y PFS: 60% 1-y DMFS: 75%	G3 dermatitis (13%) G3 mucositis (10%) G3 odynophagia (10%) G3 dysphagia (5%) G3 xerostomia (3%) G3 pain (8%) G3 ototoxicity (10%) G3 dysphagia (2%) G3 xerostomia (2%) G3 neurotoxicity (3%) G3 tracheostomy (3%) G4 ORN (3%) G5/death (2%)
Azami et al. [19] 2009–2012 Southern Tohoku Proton Therapy Center (Japan)	Concurrent intra-arterial chemotherapy (CDDP) (50%)	62	24	1-y OS: 80% 1-y LC: 80% 3-y OS: 60% 3-y LC: 60%	No ≥ G3 toxicities encountered
Yang et al. [20] 2014–2018 Shanghai Proton and Heavy Ion Center (China)	Concurrent chemotherapy (n = 6, 50%)	36	15.7	1-y OS: 67%	G4 bleeding (8%) G5 bleeding (8%)
Dale et al. [21] 2012–2016 National Center of Oncological Hadrontherapy (CNA0) (Italy)	Not specified	55	13.4	1-y CB rate: 2.7% 1-y OS: 81.5%	G5 bleeding (12%), not specified if cause of death was due to CB or tumor progression No other toxicities reported
Marucci et al. [22] 1984–2000 Harvard Cyclotron Laboratory (United States)	NR	61	59	5-y OS: 63% 5- y LR: 31% 5-y FFM: 66% 5-y survival with eye retention: 40%	9 eyes (29%) enucleated after reRT, 4 of them because of painful eye. Only 5/15 pts who had 20/200 vision before reRT maintained vision at that level
McDonald et al. [23] 2005–2012 Indiana University (United States)	NR	59	23	2-y LC: 85% 2-y OS: 80% 2-y DM: 20%	G3 laryngeal edema (6%), required permanent trach G4 ventricular obstruction, required urgent shunt placement, G3 brain necrosis (6%) G4 ischemic BS stroke (6%) G4 CSF leak/meningitis (6%) 2-y estimated late grade 3 or 4 toxicity: 19%
Lin et al. [24] 1991–1997 Loma Linda University Medical Center (United States)	Chemotherapy and/or immunotherapy was administered in (81%), either in conjunction with conventional RT, following conventional RT, or following PBT	46 (mean)	23.7 (mean)	2-y LRPFS: 50% 2-y OS: 50% 2-y LC: 50% 2-y DFS: 50%	Acute toxicities not graded G3–4 ORN (6%) G3–4 chronic ulceration nasopharynx (6%) G3 trismus (6%) G3–4 serous otitis (12%) No CNS complications observed

**Table 2.** Carbon ion reirradiation (reRT) head and neck (HN) and skull base series.

**Author [citation] study period institution (country)**	**Study type** **No. pts**	**Primary site** **histology (%)**	**Previous RT technique (%)** **median previous RT dose (range)/fractionation**	**Recurrent site** **salvage surgery before reRT (%)** **median time to reRT (mo)** **median No. previous RT treatments (range)**
Hu et al. [25] 2015–2017 Shanghai Proton and Heavy Ion Center (China)	Retrospective, single institution 75	Nasopharynx poorly differentiated or undifferentiated SCC (100%)	IMRT (96%), non-IMRT (4%) 70 Gy (66–75.75)	Nasopharynx NR 29 (11–216) NR
Jensen et al. [26] 2009–2010 Heidelberg Ion Therapy Centre (Germany)	Retrospective, single institution 15/16	Skull base (50%) Paranasal sinus (19%) Nasopharynx (12%) Posterior fossa (6%) EAC (6%) ACC (38%) Mucoepidermoid (6%) Acinic cell (6%) Chordoma (25%) Chondrosarcoma (12%) SCC (12%)	EBRT (56%) PBT (6%) CT (38%) 67 Gy (38–72)	Skull base (50%) Paranasal sinus (19%) Nasopharynx (12%) Posterior fossa (6%) EAC (6%) 0% 73 (12.2–349.6) 1
Yamazaki et al. [27] 2000–2010 (multi-institution^a^) (Japan)	Retrospective, multi-institution 17/26 CT 9/26 PBT	Nasopharynx (15%), oral cavity (8%), salivary gland (12%), sinonasal (58%), other (8%) SCC (46%) Other (54%)	EBRT or PBT (% NR) ≥ 40 Gy	Nasopharynx (15%), oral cavity (8%), salivary gland (12%), sinonasal (58%), other (8%) 9 (35%) 13 (4–92) NR
Feehan et al. [28] 1981–1990 Lawrence Berkeley Laboratory (United States)	Retrospective, single institution 11	Nasopharynx SCC (91%) Lymphoepithelioma (9%)	EBRT ± brachytherapy (100%) 70.2 Gy (61–81)	Skull base (100%) plus neck (18%) 0% NR NR
Held et al. [29] 2010–2017 Heidelberg Ion Therapy Centre (Germany)	Retrospective, single institution 229	Salivary glands (24%) Nasopharynx (23%) Paranasal sinus (21%) Oral cavity (10%) Oropharynx (6%) Hypopharynx (2%) Other (14%) ACC (54%) SCC (26%) Adeno (8%) Other (11%)	IMRT (35%) 3D-conformal (23%) IMRT + CT boost (28%) Other (6%) Unknown (8%) 67.4 Gy (36.5–84)	Salivary glands (24%) Nasopharynx (23%) Paranasal sinus (21%) Oral cavity (10%) Oropharynx (6%) Hypopharynx (2%) Other (14%) 39 (17%) 3.9 y 1 prior course (93%) 2 prior courses (7%)
Hayashi et al. [30] 2007–2016 Hospital National Institute of Radiological Sciences (Japan)	Retrospective, single institution 48	Nasal (37%), paranasal (31%), lacrimal gland/orbit (8%), nasopharynx (6%), palate (4%), SMG (2%), tongue (2%), bone of skull or cervical vertebra (8%), other (4%) Mucosal melanoma (44%) ACC (35%) Bone/soft tissue sarcoma (12%) Others (8%)	NR 57.6 Gy (48–70.4)/12–16 fractions	Paranasal (37%), nasal (19%), nasopharynx (8%), orbit (6%), cavernous sinus (6%), bone of skull or cervical vertebra (12%), other (10%) 0 % 24.2 (4.5–112.5) 1 prior course (85%) 2 prior courses (15%)
Jensen et al. [31] 2010–2013 Heidelberg Ion Therapy Centre (Germany)	Retrospective, single institution 52	NR ACC (100%)	14 pts prior CT, rest of cases not specified 66 Gy (20–115)	Paranasal (36%), base of skull/intracranial (21%), parotid (19%), SMG (6%), nasopharynx (4%), pterygopalatine fossa (4%), orbit (4%), other (6%) 7 (13%) 61 (9–620) NR
Combs et al. [32] 1997–2008 University Hospital of Heidelberg (Germany)	Retrospective, single institution 28	Skull base (64%), head and neck (18%), brain (11%), sacrum (7%) Chordoma (61%) Chondrosarcoma (11%) ACC (14%) Meningioma (11%) SCC (3%)	EBRT (65%) Gamma Knife (11%) CT (21%): 1 case was combined CT + IMRT PTB (3%) 63.4 Gy (50–79.7) for non-SRS pts	Skull base (68%), head and neck (14%), brain (11%), sacrum (7%) NR 46.7 1 prior course (89%) 2 prior courses (11%)
Gao et al. [33] 2015–2017 Shanghai Proton and Heavy Ion Center (China)	Retrospective, single institution 141	Nasopharynx (78%), nasal cavity/paranasal sinus (8%), oropharynx (3%), salivary glands (3%), larynx/hypopharynx (1%), other (3%) SCC (75%) ACC (7%) Adeno (2%) Mucoepidermoid (2%) RAdiation-induced secondary malignancy (5%) Other (4%)	IMRT (91%) SRS Gamma Knife (1%) Not recorded (8%) NR	NR 23 (16%) At least > 11 mo 1 prior course only (100%)
Guan et al. [34] 2014–2018 Shanghai Proton and Heavy Ion Center (China)	Retrospective, single institution 14/91	Skull base (93%), cervical spine (7%): numbers are for the entire cohort Chordoma (85%), chondrosarcoma (15%): numbers are for the entire cohort	SRS Gamma Knife (n = 7, 50%) SRS Cyber Knife (n = 2, 14%) Conventional RT (n = 5, 36%) NR	NR NR NR NR
Vischioni et al. [35] 2013–2016 National Center of Oncological Hadrontherapy (Italy)	Retrospective, single institution 51	Salivary gland (100%) ACC (74%) Mucoepidermoid (12%) Myoepithelial (6%) Ex-pleomorphic Adenoma (4%) Other (4%)	EBRT photon (100%) 60 Gy (24–78)	Parotid (33%), nasal cavity (10%), nasopharynx (6%), maxillary sinus (10%), ethmoid (6%), hard palate (6%), other different sites (30%) 40 (78%) 6.3 y 1 prior course (90%) 2 prior courses (10%)

Abbreviations: pts, patients; RT, radiotherapy; GTV, gross tumor volume; CTV, clinical target volume; PTV, planning target volume; OAR, organs at risk; F/u, follow-up; CT, carbon ion therapy; SCC, squamous cell carcinoma; IMRT, intensity-modulated radiation therapy; NR, not reported; IMCT, intensity-modulated carbon therapy; ENI, elective nodal irradiation; TP, docetaxel and cisplatin; GP, gemcitabine and cisplatin; CDDP, cisplatin; OS, overall survival; DSS, disease-specific survival; PFS, progression-free survival; LRFS, local recurrence–free survival; RRFS, regional recurrence–free survival; DMFS, distant metastasis–free survival; EAC, external auditory canal; ACC, adenoid cystic carcinoma; EBRT, external beam radiotherapy; PBT, proton beam therapy; max; maximum; BS, branch site; OR, XXXX; LC, local control; adeno, adenocarcinoma; 3D, 3-dimensional; ORN, osteonecrosis; SMG submandibular gland; DC, XXXX; PTB, Physikalisch-Technische Bundesanstalt; SRS, stereotactic radiosurgery; FSRT, fractionated stereotactic radiotherapy; LPFS, locoregional progression-free survival; RPFS, regional progression-free survival; DPFS, disease progression-free survival; DM, distant metastasis.

a Multi-institutions: Kyoto Prefectual University of Medicine; CyberKnife Center Soseikai General Hospital; Hyogo Ion Beam Medical Center; Fujimoto Hayasuzu Hospital; Japanese Red Cross Okayama Hospital; National Hospital Organization Osaka National Hospital; Osaka University Graduate School of Medicine; Miyakojima IGRT Clinic.

**Table 2.** Extended.

**Author [citation] study period institution (country)**	**reRT technique (%)** **median reRT dose (range)/fractionation** **treatment intent (%)**	**Median GTV (range)** **GTV–CTV margin** **CTV–PTV margin** **OAR doses Gy (range)** **nodal irradiation**	**Additional systemic therapies (%)**
Hu et al. [25] 2015–2017 Shanghai Proton and Heavy Ion Center (China)	IMCT 57.5 (50–66)/3 curative (100%)	NR GTV–CTV margin: 5 mm (limited up to 1 mm near OAR) CTV–PTV margin: 3–6 mm NR ENI not performed	Induction chemotherapy (61%) TP (25%) GP (23%) Others (13%) Concurrent chemotherapy (16%) Weekly CDDP (11%) High-dose CDDP (5%)
Jensen et al. [26] 2009–2010 Heidelberg Ion Therapy Centre (Germany)	CT alone (87%), IMRT + CT boost (6%), IMRT + PBT boost (6%) 44.8 Gy (36–72.7) curative (100%)	PTV volume 61.1 cm^3^ (9.2–284.1) CTV–PTV margin: 3 mm, did not extend into critical OAR Max cumulative dose to spinal cord <&thinsp50 Gy Max cumulative dose to BS <&thinsp60 Gy ENI not performed	Induction chemotherapy (6%)
Yamazaki et al. [27] 2000–2010 (multi-institution^a^) (Japan)	CT 57.6 (43.2–70.2) in 16 fractions/5×/wk curative (100%)	25.5 cm^3^ (2–188) GTV–CTV margin: 5 mm CTV–PTV margin: 3 mm NR NR	NR
Feehan et al. [28] 1981–1990 Lawrence Berkeley Laboratory (United States)	Heavy charged particle (helium, neon) 50.25 (31.8–62.3) curative (100%)	NR NR NR NR	10 pts received chemotherapy before, during, or after reRT; details not specified
Held et al. [29] 2010–2017 Heidelberg Ion Therapy Centre (Germany)	CT 51 (36–66)/3Gy fraction/5–6 fractions/wk curative (100%)	CTV volume 85.2 cm^3^ (6.3–710.5) PTV volume 128.9 cm^3^ (13.3–925) GTV–CTV margin: 2–5 mm CTV–PTV margin: 2–3 mm NR ENI not performed, involved lymph nodes were included in the CTV	None
Hayashi et al. [30] 2007–2016 Hospital National Institute of Radiological Sciences (Japan)	CT 54 (40–64)/8–16 fractions Curative (100%)	10.4 cm^3^ (0.5–89.5) GTV–CTV margin: 0 -5 mm CTV–PTV margin: 2 mm NR NR	No chemotherapy within 1 mo of commencing reRT
Jensen et al. [31] 2010–2013 Heidelberg Ion Therapy Centre (Germany)	CT alone (92%), IMRT + CT (8%) 51 (36–74)/3 Gy/fraction/5–6 fractions/wk curative (100%)	CTV volume 93 cm^3^ (6–618): CT alone CTV volume 334 cm^3^ (211–344): IMRT + CT GTV = CTV CTV–PTV margin: 2 mm Max cumulative dose to spinal cord <&thinsp50 Gy Max cumulative dose to BS <&thinsp60 Gy ENI not performed, in the pts who received IMRT coverage of local regional nodal levels was allowed	Not performed
Combs et al. [32] 1997–2008 University Hospital of Heidelberg (Germany)	CT (active raster scanning) alone (75%), IMRT or FSRT + CT (25%) 51 (42–60)/3 Gy per fraction (SBT) 45 Gy/3 Gy/fraction (HN tumors) Curative (100%)	NR NR NR NR	No concurrent chemotherapy performed
Gao et al. [33] 2015–2017 Shanghai Proton and Heavy Ion Center (China)	IMCT (100%) 60 (50–69)/2–3.5 Gy/fraction Curative (100%)	NR GTV–CTV margin: 3–5 mm, smaller margin allowed if close to critical OAR CTV–PTV margin: 1–3 mm NR ENI not allowed	Presalvage IMCT (45%), concurrent chemotherapy not recommended except for pts participating in clinical trial (% not specified)
Guan et al. [34] 2014–2018 Shanghai Proton and Heavy Ion Center (China)	PBT + IMCT boost (n = 6, 43%), IMCT alone (n = 8, 57%) 50Gy (PBT) + 15-18 Gy (IMCT boost), 57-69 Gy/19-23 fractions if IMCT alone Curative (100%)	37 cm^3^, not specifically reported for reRT pts GTV–CTV margin: 1–3 mm CTV–PTV margin: no > 5 mm NR NR	Not performed
Vischioni et al. [35] 2013–2016 National Center of Oncological Hadrontherapy (Italy)	CT (100%) 60 Gy (45–68.8)/3–5 Gy fraction/4×/wk Curative (100%)	28.58 cm^3^ (1.75–205.54) GTV–CTV margin: 0–5 mm CTV–PTV margin: 2 mm NR NR	NR

**Table 2.** Extended.

** Author [citation] study period institution (country)**	**Median age, y**	**Median F/u (mo)**	**Clinical outcomes**	**CT acute toxicities, G (%)** **CT late toxicities, G (%)**
Hu et al. [25] 2015–2017 Shanghai Proton and Heavy Ion Center (China)	48	15.4	1-y OS: 98.1% 1-y DSS: 98.1% 1-y PFS: 82.2% 1-y LRFS: 86.6% 1-y RRFS: 97.9% 1-y DMFS: 96.2%	No ≥ G2 acute toxicities ≥ G3 mucositis (9%) ≥ G3 brain necrosis (1%) ≥ G3 xerostomia (1%)
Jensen et al. [26] 2009–2010 Heidelberg Ion Therapy Centre (Germany)	51	4	OR rate: 53% 8 weeks post RT (non-chordoma/chondrosarcoma) 4/5 pts chordoma/chondrosarcoma with no signs of progression	No ≥ G3 acute toxicities No late toxicities reported due to short F/u
Yamazaki et al. [27] 2000–2010 (multi-institution^a^) (Japan)	55	8	1-y OS: 68% 1-y LC: 67%	G3 nerve palsy (8%) G3 mucosal ulceration (8%) G3 skin ulceration (4%) G4 visual disturbance (8%) G4 soft tissue necrosis (4%) G5 bleeding (8%) G5 ORN (4%) G5 soft tissue necrosis (4%)
Feehan et al. [28] 1981–1990 Lawrence Berkeley Laboratory (United States)	48	28.1	3-y OS: 59% 5-y OS: 31% LC: 45%	G2–3 brain necrosis (25%) G3 trismus (6%) G3 visual disturbance (6%) G3 hypopituitarism (6%) G4 bleeding (6%) No G5 toxicities reported
Held et al. [29] 2010–2017 Heidelberg Ion Therapy Centre (Germany)	NR	28.5	Median PFS: 24.2 mo Median OS: 26.1 mo	G3 dysphagia (1.3%) G3 fistula (0.4%) G3 impaired hearing (0.4%) G4 laryngeal edema (0.9%) G3 brain necrosis (4%) G3 impaired hearing (4%) G3 optic nerve (1.6%) G3 fistula (0.8%) G3 ORN (0.8%) G4 optic nerve (1.6%) G4 brain necrosis (0.8%) G4 bleeding (0.8%)
Hayashi et al. [30] 2007–2016 Hospital National Institute of Radiological Sciences (Japan)	56.5 at initial irradiation	27.1	2-y LC: 40.5% 2-y LRC: 33.5% 2-y PFS: 29.4% 2-y OS: 59.6%	G3 mucositis (8%) G3 dermatitis (2%) G3-4 brain necrosis (4%) G5 brain necrosis (2%) G3–4 optic nerve (23%) G3 cataract (2%) G3 trismus (2%) G3 dysphagia (2%) G4 arterial injury (2%)
Jensen et al. [31] 2010–2013 Heidelberg Ion Therapy Centre (Germany)	54	14	1-y LC: 70% 1-y OS: 82% 1-y DC: 73%	No acute ≥ G3 toxicity G3 dysphagia (2%) G3 brain necrosis (4%) G3 ORN (6%) G4 bleeding (4%)
Combs et al. [32] 1997–2008 University Hospital of Heidelberg (Germany)	<&thinsp65 (86%) ≥ 65 (14%)	41	2-y LC: 92% (SBT) 3-y LC: 64% (SBT) 2-y OS: 86% (SBT) 5-y OS: 43% (SBT) Median LPFS 24 mo (HN tumors), all HN pts death on last F/u	No ≥ G3 complications reported for skull base or head and neck tumors treated
Gao et al. [33] 2015–2017 Shanghai Proton and Heavy Ion Center (China)	49	14.7	1-y OS: 96% 1-y DSS: 96% 1-y LPFS: 85% 1-y RPFS: 98% 1-y DPFS: 96%	≥ G3 bleeding (1%) ≥ G3 mucosal necrosis (7%) with 4 pts dying of secondary bleeding, ≥ G3 brain necrosis (1%) ≥ G3 xerostomia (1%) ≥ G3 cranial neuropathy (2%)
Guan et al. [34] 2014–2018 Shanghai Proton and Heavy Ion Center (China)	38	28	At time of analysis (n = 7), 50% of reRT pts had died of uncontrolled local disease 2-y OS: 50% (reRT)	Not specifically reported for reRT pts
Vischioni et al. [35] 2013–2016 National Center of Oncological Hadrontherapy (Italy)	60	19	1-y PFS: 72% 2-y PFS: 52% 1-y OS: 90% 2-y OS: 64% At last F/u LC: 41%, and DM rate: 33%	G3 acute toxicity (4%), toxicity not clearly specified G3 late toxicity (17%), toxicity not clearly specified in all cases G3 visual deficit (6%) G3 neuropathy (2%) G3 trismus (8%)

Median age for the cohort of patients treated with proton therapy reRT was 57.9 years (range, 36.0 – 68.0 years), and 50.3 years (range, 38.0 – 60.0 years) for the carbon ion reRT group. These were evaluated on a per-study basis, as also for the RT doses and time intervals between RT courses reported below. The median previous RT dose was 64.5 Gy (range, 55.0 – 75.2 Gy, data available for 12 of the 15 studies) for the proton reRT studies, and 64.5 Gy (range, 57.6 – 70.0 Gy, data available for 7 of the 10 studies) for the carbon-ion reRT series. The median time interval between the initial RT and proton reRT course was 36.7 months (range, 24.7 – 54.0 months, data available for 9 of the 15 studies), and 46.1 months (range, 13.0 – 75.5 months, data available for 8 of the 10 studies) for the group of patients that subsequently received carbon ion reRT. The median proton therapy reRT dose was 64.5 Gy (RBE 1.1) (range, 50.0 – 75.6 Gy ), (data available for 14 of the 15 studies), while the median carbon ion therapy reRT dose was 53.8 Gy (RBE 2.5 - 3) (range, 44.8 – 60.0 Gy ); (data available for 9 of the 10 studies). Passive scattering/uniform scanning was the more frequently employed proton reRT technique in 11 of the 15 (73%) studies, and active scanning technique was used for carbon-ion reRT in 7 (70%) of the 10 reported series. Regarding additional systemic therapies, induction and/or concurrent chemotherapy was administered to 232 (53%) of the patients that received a course of proton reRT, and to 122 (18%) of reRT carbon ion patients. Cisplatin (CDDP) was the most commonly administered systemic agent.

With a median follow up of 23 months, reRT achieved 2-year local control (LC) rates ranging from 50% to 86% for proton reRT, and 41% to 92% for carbon ion reRT with a median follow up of 19 months. The 2-year overall survival rates for proton and carbon ion reRT ranged from 33% to 80% and 50% to 86% respectively. By head and neck subsite, the LC rates for sinonasal carcinomas ranged from 59 to 77%; nasopharyngeal carcinoma, 50% to 80%; and for salivary gland tumors, 60% to 86% with proton-beam reRT. For carbon-ion reRT, the LC rates for sinonasal carcinomas ranged from 41% to 67%; nasopharyngeal carcinoma, 45 to 85%; and 41% to 92% for salivary gland tumors. With respect to OS, the rates for sinonasal, nasopharyngeal carcinomas, and salivary gland tumors were 57% to 76%, 33% to 74%, and 57% to 80% with proton beam reRT, and 60% to 70%, 59 to 68%, and 64 to 82% with carbon-ion reRT.

In regard to dosimetric analysis, only 3 (20%) of the 15 proton reRT series, and none of the carbon ion reRT series, reported a detailed analysis of the employed constraints and delivered doses for the organs at risk. There was noted to be significant variability across series in the size of the tumor volumes treated, the margins employed for gross tumor volume (GTV) to the clinical target volume (CTV) expansion, and CTV to the planning target volume (PTV) expansion (Tables 1 and 2). In all series, elective nodal irradiation (ENI) was not routinely performed. With respect to associated treatment toxicities, the rates for acute ≥ grade 3 toxicities ranged from 1% to 35%, with dysphagia, mucositis and radiation dermatitis being the most frequent, and the rates for late grade ≥3 toxicities ranged up to 37% for protons and up to 35% for carbons, with brain necrosis, ototoxicity, visual deficits, and bleeding were most commonly reported. There was a total of 16 cases of grade 5 reported toxicities for all treated patients (n= 16/1118, 1.4%) with fatal bleeding as the leading cause (Tables 1 and 2).

## Discussion

Locoregional recurrences remain a common pattern of failure, morbidity and death in head and neck cancer patients [36 – 38]. Even with multimodality therapy, patients typically have poor oncologic outcomes, with increased severe treatment related toxicities [39 - 41].

Management of patients suffering from recurrent head and neck cancer is typically very challenging with no single treatment algorithm appropriate for all patients. Recurrent head and neck cancers are a heterogeneous group of patients, involving a number of different histologies and disease subsites. When evaluating a patient for reRT, a multidisciplinary evaluation that considers patient age, baseline comorbidities, performance status, histology, tumor biology, anatomic location, prior treatment constraints/toxicities, time interval since prior RT course, organ dysfunction (tracheostomy, feeding tube dependency), and patient goals and expectations is essential to determine which interventions may be the most appropriate [42]. To our knowledge this is the first comprehensive systematic review of the current existing data on the use of charged particle reRT for the definitive management of recurrent or secondary skull base and head and neck malignancies.

Published literature on the use of photon radiotherapy reRT for recurrent or second primary skull base and head and neck cancers have shown significant variability in the reported outcomes and toxicities, depending on photon therapy modality used (3D-conformal, IMRT, brachytherapy, IORT, SBRT), delivered dose and additional therapies employed, with 2-yr LC rates ranging from 19% – 67% and 2-yr OS rates ranging from 11% – 81% and with ≥ grade 3 toxicities up to 59%, and in some series risk of grade 5 toxicity (often secondary to carotid rupture) in up to 24% [7, 39 – 41, 43 – 54]. Our analysis demonstrated 2-year local control rates in the range of 50% to 86% for proton, and 41% to 92% for carbon ion reRT. The 2-yr OS rates for proton and carbon ion reRT ranged from 33% to 80% and 50% to 86% respectively. In regards to treatment related late ≥ grade 3 toxicities this ranged between 0% to 37% overall, with only sixteen grade 5 reported toxicities of the 1118 analyzed patients for a 1.4% rate (Tables 1 and 2).

Table 3 summarizes reported clinical outcomes by reRT modality [7, 39 – 41, 43 – 54]. Although no conclusive comparisons can be drawn from these data given inherent differences in patient selection among the studies, results are suggestive that potentially more favorable LC and toxicity outcomes could be realized with charged particle therapy in properly selected patients. Of note, SBRT reRT data which do not include concurrent systemic therapy, demonstrate lowest rates of severe late toxicity likely due to smaller treatment target volumes in these studies, yet with largely similar LC compared to other photon studies. A proposition to utilize proton SBRT with concurrent immunotherapy to enhance LC, while limiting severe toxicity is currently under investigation [ClinicalTrials.gov Identifier: NCT03539198]. More advanced charged particle delivery techniques such as intensity-modulated proton beam therapy (IMPT), and intensity-modulated carbon ion therapy may further improve the therapeutic window for reirradiation. This may enable treatment of larger recurrent tumors with higher doses which would otherwise be more difficult to achieve with photon-based SBRT reRT approaches, due to greater collateral dose to organs-at-risk when treating larger treatment volumes [55].

**Table 3.** Reported results by reirradiation modality.

**Treatment [source]**	**2-year LC, %**	**2-year OS, %**	**Grade 3+ late toxicity, %**
Proton therapy [current study]	50–86	33–80	0–33
Carbon ion [current study]	41–92	59–82	0–37
SBRT [45–47]	30–58	14–58	0–18
IMRT [7, 48–51]	19–67	12–68	15–48
3DCRT [39–41, 53, 54]	20–37	11–81	21–59
BT (HDR and LDR) [52]	31–69	13–43	4–36

Abbreviations: LC, local control; OS, overall survival; SBRT, stereotactic body radiotherapy; IMRT, intensity-modulated radiotherapy; 3DCRT, 3-dimensional conformal radiotherapy; BT, brachytherapy; HDR, high-dose rate; LDR, low-dose rate.

This systematic review is not exempt from several limitations including most of the evaluated series were retrospective from single institutions, with significant variability in patient selection, recurrent disease sites, histologies, treatment technique, doses and fractionation employed, and the reported toxicities and outcomes. In addition, there were also statistical limitations and biases of the analysis due to the inherent heterogeneity of the reRT reports and lack of availability critical data within some of the reRT series, including prior RT doses, median interval times between RT courses and not having well defined study endpoints that could impact on the interpretation of the outcomes. There are no randomized data comparing outcomes with photons (IMRT, SBRT) versus charged particle reRT (protons, carbon ions). With the continuous increase in availability of centers with capability to deliver charged particle radiotherapy we can anticipate more data will emerge, and help to further elucidate the potential clinical benefits of these treatment modalities.

Efforts should continue to be made to design clinical trials that can collect robust data on the use of charged particle reRT, and would advance the management of these patients resulting in a better understanding on the selection of patient candidates for this treatment paradigm. A published patient selection RPA classification may facilitate patient stratification in future studies using charged particle reRT, to inform best study design and treatment strategies [56]. Despite the complexity in the management of these malignancies, the current accumulated information on the use of charged particle reRT for recurrent or second primary skull base and head and neck cancers is encouraging and may advocate the potential clinical advantages of the use of charged particle in this setting.

## Conclusions

Based on the current available data, curative intent head and neck reRT with charged particle radiotherapy is feasible and well tolerated in the majority of patients, with the potential to improve oncologic and toxicity outcomes in well-selected cases. Prospective studies of patients reporting in more depth oncologic outcomes and dosimetric treatment planning data are necessary to further validate these findings.
